# Association Between Homocysteine, Vitamin B12, Folate and Migraine: An Updated Systematic Review and Meta-Analysis

**DOI:** 10.3390/brainsci16020218

**Published:** 2026-02-11

**Authors:** Islamia Ibrahim Ahmed Omer, Eman A. Kubbara, Tassneem Awad Hajali, Nouralsalhin A. Alaagib, Hamdan Z. Hamdan

**Affiliations:** 1 Department of Clinical Science, College of Medicine, Sulaiman AlRajhi University, AlBukayriah 51941, Qassim, Saudi Arabia; i.omer@sr.edu.sa (I.I.A.O.); t.hajali@sr.edu.sa (T.A.H.); 2 Clinical Biochemistry Department, Faculty of Medicine, Rabigh Branch, King Abdulaziz University, Rabigh 21911, Makkah, Saudi Arabia; eadm@kau.edu.sa; 3 Department of Basic Science, College of Medicine, Sulaiman AlRajhi University, AlBukayriyah 51941, Qassim, Saudi Arabia; n.alaagib@sr.edu.sa; 4 Department of Pathology, College of Medicine, Qassim University, Buraidah 51452, Qassim, Saudi Arabia

**Keywords:** homocysteine, migraine, vitamin B12, folate, adult, pediatrics, children

## Abstract

**Highlights:**

**What are the main findings?**

- Adults and children with migraines were found to have higher levels of homocysteine in their blood and lower levels of vitamin B12 and folate than people without migraines.
- These biochemical differences persist across geographic regions and laboratory methods, supporting a potential metabolic link between migraine and one-carbon metabolism.

**What are the implications of the main findings?**

- Clinicians may consider evaluating homocysteine, vitamin B12, and folate levels in migraine patients, as these factors may influence migraine risk and severity.
- Implementing routine assessment and targeted correction of these markers could provide a practical adjunct to migraine prevention and management.

**Abstract:**

**Background**: Migraine is a neurovascular disease; its pathogenesis has been linked to higher levels of homocysteine (Hcy) and/or deficiencies in vitamins (vitamin B12 and folate). However, previously published studies remained inconclusive. Therefore, the aim of this study is to review the literature to update the current evidence and clarify the association between Hcy, vitamin B12, folate and migraine in adult and pediatric patients. **Methods**: We searched the databases PubMed, ScienceDirect, Google Scholar, and the Cochrane Library for articles that investigated levels of Hcy, B12, and folate in association with migraine headaches, since inception through December 2025. The package “meta” in R software was used to calculate the standardized mean difference (SMD) of Hcy, B12 and folate in cases of migraine and compared with non-migraine controls. **Results**: A total of 17 studies (15 case–control and 2 cross-sectional) investigated the levels of Hcy, encompassing 1549 cases of migraine and 1824 non-migraine controls. The random effect model showed a significantly higher SMD for Hcy in migraine cases compared with non-migraine controls [SMD = 0.48, 95% CI (0.12; 0.83); *p* < 0.01; I^2^ = 91.0%]. Stratification analysis showed the same trends in a group of studies that was conducted in European countries [SMD = 0.29; 95% CI (0.04; 0.54); *p* = 0.02; I^2^ = 87.0%] and group of studies that used analytical methods other than immunoassays [SMD = 0.28; 95% CI (0.08; 0.49); *p* < 0.001; I^2^ = 84.0%]. Meta-regression results showed that only the year of publication had a significant positive effect [estimation coefficient = 0.087; *p* = 0.017]. Serum levels of vitamin B12 [16 studies included 1330 cases vs. 1533 controls, SMD = −0.36, 95% CI (−0.62; −0.10); *p* < 0.01; I^2^ = 92.1%] and folate [10 studies included with 793 cases vs. 1011 controls, SMD = −0.25 [−0.47; −0.04], *p* = 0.02; I^2^ = 77.3%] were found to be significantly lower in migraine cases compared with non-migraine controls, respectively. **Conclusions:** Adult and pediatric patients with migraine had elevated Hcy levels and lower vitamin B12 and folate levels. Clinicians may check and correct for Hcy, vitamin B12, and folate levels as prophylactic and therapeutic interventions for migraine. Further studies with a longitudinal design are needed to establish a causal relationship.

## 1. Introduction

Migraine is a recurrent neurological disorder characterized by moderate to severe headaches, often accompanied by reversible neurological and systemic symptoms [1,2]. The International Headache Society classifies migraine into two categories: migraine with aura (MA) and migraine without aura (Society HCS 2004). Migraine attacks may present with cranial autonomic symptoms and cutaneous allodynia (Headache Classification, 2013).

Globally, migraine was the second leading cause of disability-adjusted life years (DALYs), the sum of years of life lost (YLLs) and years lived with disability (YLDs) due to neurological disorders in 2016 [3]. Migraine significantly affects individuals’ economic status, family relationships, and participation in work and school activities [4]. The prevalence of migraine varies both between and within countries [5]. The global age-standardized prevalence of migraine increased by approximately 1.7% from 1990 to 2019 [6]. Among adults, migraine prevalence ranges from 2.6% to 21.7%, with an average of approximately 12% [5]. Migraine is also prevalent in children, affecting 7.7% to 9.1% of children and adolescents [7]. In individuals under 18 years, migraine is more frequently bilateral compared to adults. Children with migraine often experience reduced quality of life in physical, socio-economic, and school functioning compared to their healthy peers [8].

Multiple biological factors, including hormonal, genetic, and metabolic disorders, as well as psychiatric and psychological factors, are considered risk factors for migraine [9]. Several studies have proposed that elevated plasma homocysteine (Hcy) and hyperhomocysteinemia (HHcy) contribute to migraine pathophysiology [10]. Recent findings indicate that homocysteine increases the sensitivity of peripheral migraine mechanisms [11]. Hcy is metabolized to cystathionine or remethylated to methionine by methylenetetrahydrofolate reductase (MTHFR) [12]. Certain MTHFR polymorphisms reduce Hcy remethylation, resulting in HHcy. The metabolism of homocysteine primarily requires vitamins B12, folate, and, to a lesser extent, B6. Nutritional deficiencies in these cofactors can lead to hypomethylation, triggering HHcy and potentially contributing to migraine occurrence [12,13,14]. Supplementation with vitamin B12 and folate has been shown to significantly reduce elevated Hcy levels and migraine frequency [15]. Thus, maintaining levels of these vitamins may serve as a useful prophylactic strategy for preventing migraine [16]. Recent evidence suggests that controlling homocysteine levels may help reduce migraine attacks in humans [11].

Conversely, some studies have reported an insignificant association between Hcy levels and migraine severity [17] and insignificant differences in Hcy levels between migraine patients and control subjects [18,19]. Other studies have found an insignificant association between folic acid and vitamin B12 supplementation and migraine outcomes [20,21]. Although a previous meta-analysis was published [10], several subsequent studies have reported conflicting findings [22]. Therefore, the current systematic review and meta-analysis aimed to update the current knowledge and resynthesize the evidence on the association between Hcy, B12 and folate levels with migraine. This update will emphasize our understanding of the potential roles of Hcy, folate and B12 in association with migraine. Such understanding will be helpful for care providers, as monitoring Hcy levels and the use of lowering vitamins in migraine patients may improve clinical practice. Therefore, this update is worthwhile.

## 2. Materials and Methods

### 2.1. Study Protocol and Search Strategy

The updated version of the Preferred Reporting Items for Systematic Reviews and Meta-Analyses (PRISMA) guideline was followed in this systematic review and meta-analysis, as shown in the PRISMA checklist (Table S1). The systematic review and meta-analysis protocol was prospectively registered in PROSPERO registry, with the registration number CRD420261287446. We searched for eligible published articles that measured homocysteine, vitamin B12 and folate levels in patients diagnosed with migraine and compared them with those in non-migraine control subjects across databases, including PubMed, ScienceDirect, Google Scholar, and Cochrane Library. We used MeSH and non-MeSH terms, along with the Boolean operators [OR] and [AND], to retrieve eligible studies; see Table S2 for the full list of keywords and the search strategy. Briefly, the search strategy is based on the Population Intervention Comparison Outcome and Study design (PICOS) format, as seen in Table 1:

Three reviewer investigators (EAK, TAH, and IIAO) conducted the initial search and screening of articles independently. Titles and abstracts were reviewed to decide whether to obtain the full text of each article or to reject it from the meta-analysis. The full texts of eligible articles were retrieved, and their reference lists were reviewed for additional possible studies. Each reviewer investigator decided which articles to include in the meta-analysis based on the inclusion and exclusion criteria. Any disputes were resolved by discussion with a judging reviewer (HZH).

### 2.2. Inclusion Criteria

Studies were included for this meta-analysis if they assessed serum or plasma levels of homocysteine, vitamin B12, or folate in patients with migraine and compared these measurements with those of non-migraine control participants; reported biomarker concentrations as means with standard deviations or provided data convertible to means (SD); and used case–control, cohort, or cross-sectional study designs. No restrictions were imposed on publication language.

### 2.3. Exclusion Criteria

Studies were excluded if they did not compare the levels of either homocysteine, vitamin B12 or folate with a non-migraine control; reported the levels of these metabolites in non-convertible format to mean (SD); included patients supplemented with vitamin B12 or folic acids; or were published in one of the following formats: letter to the editor, conference proceedings, case reports, reviews, and experimental animal studies.

### 2.4. Definition of the Outcome of the Interest

The primary outcome of the current meta-analysis is to determine the association between homocysteine, vitamin B12, and folate levels and migraine headaches in adult and pediatric populations. To assess this association, we calculated standardized mean differences (SMDs) in homocysteine, vitamin B12, and folate levels between migraine cases and non-migraine control subjects. Migraine headaches are diagnosed according to the criteria of the International Classification of Headache Disorders [23], a guideline defined by the International Headache Society.

### 2.5. Assessment of Risk of Bias

The quality of each included study was assessed using the Newcastle–Ottawa Scale (NOS). The standard NOS was used for case–control and cohort studies, while a modified version was used for cross-sectional studies. This tool evaluates study quality in three main areas: participant selection, group comparability, and outcome assessment. Quality was rated on a star-based system, with a maximum of nine stars. Studies with ≥7 stars were considered high quality, those with 3 to <7 stars as moderate, and those with fewer than 3 stars as low quality.

### 2.6. Data Extraction

After retrieving the full articles, three reviewer investigators extracted the following data from each article: the last name of the 1st author, year of publication, study country, levels of homocysteine, vitamin B12, and folate in migraine patients and the non-migraine control, diagnostic criteria used for diagnosing migraine, laboratory methods for determining homocysteine, vitamin B12 and folate, study design, and sample size. All these data were recorded in a Microsoft Excel sheet and double-checked before performing the meta-analysis.

If the levels of homocysteine, vitamin B12, and folate were presented in formats other than mean (SD), we calculated the mean (SD) using previously described methods [24,25].

### 2.7. Evidence Certainty Using the GRADEpro GDT Tool

The certainty of the synthesized evidence was evaluated using the GRADEpro GDT online platform [26], which appraises evidence quality based on the risk of publication bias, result inconsistency, indirectness of evidence, and imprecision.

### 2.8. Statistical Analysis

Statistical analyses were conducted using R software version 4.4.0 (R Foundation for Statistical Computing, Vienna, Austria), as described previously [27,28]. Briefly, the meta package (version 7.0) estimated the pooled standardized mean difference (SMD) for homocysteine, vitamin B12, and folate levels between migraine and non-migraine groups using the metacont function. Study heterogeneity was assessed using Cochran’s Q test and the Higgins inconsistency index (I^2^). Heterogeneity was considered present when Cochran’s Q yielded *p* < 0.01, and I^2^ exceeded 50%. Under these conditions, a random-effects model was applied; otherwise, a fixed-effects model was used. A cumulative meta-analysis was conducted to show how the effect size had evolved over time by adding one study at each step. The metacum function in the meta package was used after sorting the studies chronologically by publication year, and a cumulative forest plot was created. Subgroup analyses were planned before analysis to explore sources of heterogeneity by geographic region, study design, included population (adult vs. children), and metabolite determination methods. Meta-regression analyses identified covariates influencing the pooled SMDs for homocysteine, vitamin B12, and folate. Sensitivity analyses evaluated the impact of individual studies on effect size and heterogeneity. Publication bias was assessed qualitatively by funnel plot inspection and quantitatively by Egger’s and Begg’s tests. Statistical significance was defined as a two-sided *p*-value < 0.05.

## 3. Results

### 3.1. Study Selection

**Figure 1:**
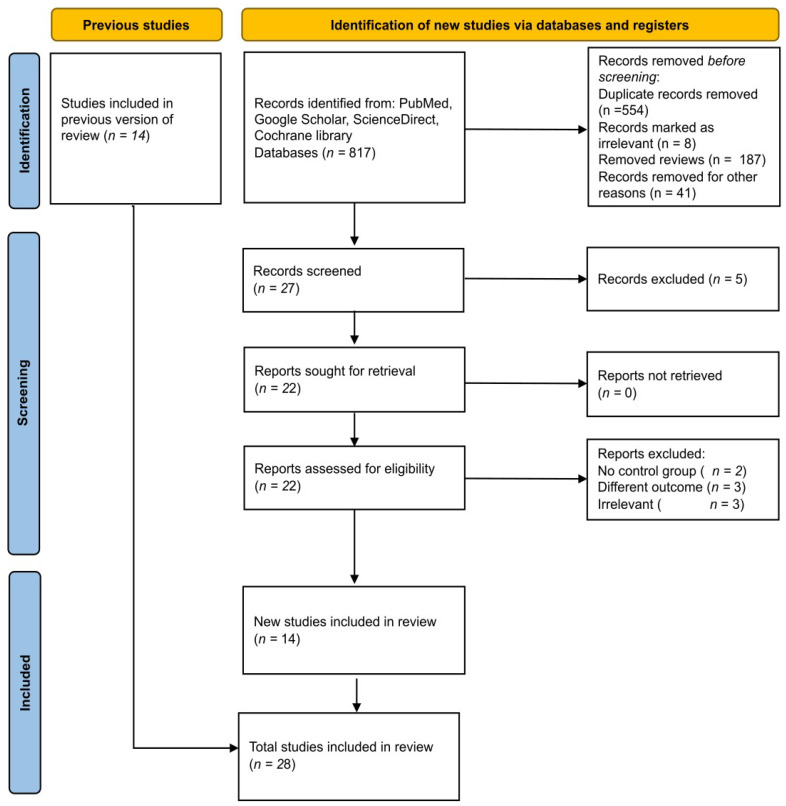
Study flowchart demonstrating study selection.

The initial search for eligible articles across all databases yielded 817 records. After the removal of duplicates and exclusion of case reports, animal studies, editorials, proceedings, and reviews, 27 records remained eligible for screening. Thereafter, the reviewers assess the title and abstract of each article carefully before retrieving the full text of 22 articles. Following a full-text evaluation, an additional eight articles were excluded due to the absence of controls, assessment of other outcomes, and irrelevance. Finally, 14 newly identified studies, along with 14 studies retrieved from the previous review, were included in the final meta-analysis (see Figure 1).

### 3.2. Features of Selected Studies

#### 3.2.1. Homocysteine Studies

We selected 17 published articles that measured homocysteine levels in migraine patients and non-migraine controls and included them in the meta-analysis. The meta-analysis included 1549 cases of migraine and 1824 controls. Geographically, ten studies were conducted in Europe, with six studies in Turkey [29,30,31,32,33,34], three in Italy [19,35,36] and one in Spain [37]. Six studies were conducted on the Asian continent, which included two studies in India [17,38], one in Kuwait [39], one in Pakistan [18], one in Iran [40], and one in South Korea [41], while only one study was conducted in the United States of America [42]. Two studies used a cross-sectional design [38,39], while the remaining studies used a case–control design. All selected studies were conducted in the adult population, whereas Bottini et al.’s study [35] was conducted in children, and İPÇİOĞLU et al. [31] studied only female cases and controls. Three studies investigated the levels of homocysteine in migraine patients with aura and migraine without aura [30,37,38]; see Table 2.

#### 3.2.2. Vitamin B12 Studies

Regarding studies investigating B12 levels, we selected 16 studies comprising 1330 migraineurs and 1533 non-migraineur controls. Twelve studies were conducted in Europe, with ten in Turkey [22,30,31,32,43,44,45,46,47,48] and two in Italy [19,35]. Three studies were conducted in Asia, one in Iran [49], one in South Korea [41], and one in Pakistan [50]. In addition, only one study was conducted in Africa (Egypt) [13]. All studies followed a case–control design and were conducted in the adult population, except for two that investigated children [35,46]; see Table 2.

**Table 2:** Characteristics of the studies included in the meta-analysis of homocysteine, vitamin B12, and folate levels in migraine cases.

Homocysteine Studies	Country	Study Design	Sample Size Cases:Controls		Levels of Homocysteine in Cases Mean (SD)	Levels of Homocysteine in Controls Mean (SD)	Diagnostic Criteria	Homocysteine Assay	NOS
Al_Hashel et al., 2022 [39]	Kuwait	Cross-sectional	60	20	7.80 (4.48)	6.01 (1.11)	ICHD-III	Other methods	8
Avci et al., 2019 [29]	Turkey	Case–control	74	74	8.12 (2.50)	7.96 (1.90)	ICHD-III	Immunoassay	7
Bahadir (A) et al., 2013 [30]	Turkey	Case–control	78	107	15.30 (3.70)	13.57 (3.93)	ICHD-II	HPLC	6
Bahadir (B) et al., 2013 [30]	Turkey	Case–control	72	107	14.99 (4.68)	13.57 (3.93)	ICHD-II		6
Bokhari et al., 2010 [18]	Pakistan	Case–control	27	32	24.38 (0.26)	23.43 (5.61)	IHS criteria	EIA	7
Bottini et al., 2006 [35]	Italy	Case–control	45	45	8.71 (1.20)	8.05 (2.2)	ICHD-II	HPLC	7
Chowdary (A) et al., 2022 [38]	India	Cross-sectional	30	30	30.87 (7.48)	9.57 (2.13)	ICHD-II	ELISA	7
Chowdary (B) et al., 2022 [38]	India	Cross-sectional	30	30	12.82 (2.56)	9.57 (2.13)	ICHD-II		7
Eroz et al., 2014 [32]	Turkey	Case–control	176	123	15.32 (4.29)	13.631 (3.93)	ICHD-II	HPLC	6
Ferraris et al., 2003 [19]	Italy	Case–control	34	36	11.08 (3.57)	12.8 (6.01)	IHS	Immunoassay	7
Gavgani et al., 2012 [40]	Iran	Case–control	65	65	14.49 (5.03)	10.92 (4.68)	ICHD-II	Immunoassay	7
İPÇİOĞLU et al., 2008 [31]	Turkey	Case–control	50	46	10.96 (4.13)	8.67 (1.99)	IHS	HPLC	6
Kharb et al., 2017 [17]	India	Case–control	30	30	10.80 (3.40)	9.3 (2.65)	IHS	Other methods	8
Moschiano et al., 2008 [36]	Italy	Case–control	136	117	12.31 (11.02)	9.86 (3.71)	ICHD-II	Immunoassay	8
Oterino (A) et al., 2010 [37]	Spain	Case–control	199	310	11.02 (4.10)	10.62 (1.10)	IHS	HPLC	7
Oterino (B) et al., 2010 [37]	Spain	Case–control	199	310	9.94 (2.40)	10.62 (1.10)	IHS	HPLC	7
Sari et al., 2011 [34]	Turkey	Case–control	66	66	10.91 (5.63)	10.00 (3.18)	ICHD-II	Other methods	7
Seo et al., 2015 [41]	Korea	Case–control	73	121	10.24 (5.69)	9.15 (4.46)	IHS	Immunoassay	7
Tietjen et al., 2009 [42]	USA	Case–control	50	125	6.33 (0.13)	6.46 (0.20)	ICHD-II	Other methods	6
Varol et al., 2015 [33]	Turkey	Case–control	55	30	8.62 (0.99)	7.31 (0.64)	ICHD-II	Immunoassay	7
**B12 studies**	Country	Study Design	Sample Size Cases:Controls		Levels of B12 in Cases Mean (SD)	Levels of B12 in Controls Mean (SD)	Diagnostic Criteria	B12 Assay	NOS
Abdelsadek et al., 2025 [13]	Egypt	Case–control	90	90	243.9 (124.8)	302.6 (143.7)	ICHD-III	ELISA	8
Acar et al., 2011 [43]	Turkey	Case–control	51	28	215.6 (133.7)	289.9 (12.0)	ICHD-II	Immunoassay	7
Atik et al., 2021 [44]	Turkey	Cross-sectional	54	51	322.1 (123.2)	439.1 (152.7)	ICHD-II	Other methods	7
Ayanoğlu et al., 2021 [45]	Turkey	Case–control	46	103	313.0 (131.0)	405.0 (238.0)	ICHD-III	Immunoassay	7
Aydin et al., 2020 [46]	Turkey	Case–control	65	87	196.4 (95.5)	240.0 (105.2)	ICHD-III	Other methods	6
Bahadir (A) et al., 2013 [30]	Turkey	Case–control	78	107	341.0 (279.0)	330.5 (236.0)	ICHD-II	HPLC	6
Bahadir (B) et al., 2013 [30]	Turkey	Case–control	72	107	344.4 (268.6)	330.5 (236.0)	ICHD-II		6
Bottini et al., 2006 [35]	Italy	Case–control	45	45	624.0 (255.0)	556.0 (207.0)	ICHD-II	HPLC	7
Eroz et al., 2014 [32]	Turkey	Case–control	176	123	317.5 (197.4)	304.5 (143.8)	ICHD-II	HPLC	6
Ferraris et al., 2003 [19]	Italy	Case–control	34	36	374.0 (194.0)	350.0 (143.0)	IHS	Immunoassay	7
İPÇİOĞLU et al., 2008 [31]	Turkey	Case–control	50	46	280.0 (86.0)	275.0 (75.0)	IHS	HPLC	6
Kılıç et al., 2026 [47]	Turkey	Case–control	95	260	181.3 (53.0)	393.4 (126.6)	ICHD	Other methods	6
Özçora et al., 2022 [48]	Turkey	Case–control	54	64	356.5 (129.4)	344.0 (139.0)	ICHD-III	Immunoassay	6
Ozek et al., 2022 [22]	Turkey	Case–control	127	45	227.3 (104.7)	278.4 (149.8)	ICHD-III	Immunoassay	7
Seo et al., 2015 [41]	Korea	Case–control	73	121	601.0 (193.0)	683.0 (591.0)	ICHD-I	Immunoassay	7
Siyal et al., 2024 [50]	Pakistan	Case–control	150	150	210.5 (72.3)	280.6 (78.4)	ICHD-III	Immunoassay	5
Togha et al., 2019 [49]	Iran	Case–control	70	70	512.0 (30.0)	667.0 (351.0)	ICHD-III	ELISA	7
**Folate studies**	Country	Study Design	Sample Size Cases:Controls		Levels of Folate in Cases Mean (SD)	Levels of Folate in Controls Mean (SD)	Diagnostic Criteria	Folate Assay	NOS
Acar et al., 2011 [43]	Turkey	Case–control	51	28	6.74 (4.31)	8.47 (1.85)	ICHD-II	Immunoassay	7
Atik et al., 2021 [44]	Turkey	Cross-sectional	54	51	8.85 (3.49)	7.24 (7.17)	ICHD-II	Other methods	7
Aydin et al., 2020 [46]	Turkey	Case–control	65	87	5.44 (1.70)	8.18 (4.41)	ICHD-III	Other methods	6
Bahadir (A) et al., 2013 [30]	Turkey	Case–control	78	107	9.72 (2.88)	9.76 (3.47)	ICHD-II	HPLC	6
Bahadir (B) et al., 2013 [30]	Turkey	Case–control	72	107	9.97 (3.42)	9.76 (3.47)	ICHD-II		6
Bottini et al., 2006 [35]	Italy	Case–control	45	45	5.84 (2.61)	7.50 (2.11)	ICHD-II	HPLC	7
Eroz et al., 2014 [32]	Turkey	Case–control	176	123	9.71 (3.14)	9.79 (3.46)	ICHD-II	HPLC	6
Ferraris et al., 2003 [19]	Italy	Case–control	34	36	5.93 (3.37)	8.0 (4.51)	IHS	Immunoassay	7
İPÇİOĞLU et al., 2008 [31]	Turkey	Case–control	50	46	8.10 (2.60)	8.11 (3.30)	IHS	HPLC	6
Kılıç et al., 2026 [47]	Turkey	Case–control	95	260	8.82 (4.04)	11.49 (4.65)	ICHD	Other methods	6
Seo et al., 2015 [41]	Korea	Case–control	73	121	9.41 (4.30)	10.61 (8.21)	ICHD-I	Immunoassay	7

(A): Includes migraine cases with aura; (B): includes migraine cases without aura.

#### 3.2.3. Folate Studies

We have included ten studies that investigated folate levels in cases of migraine and controls. These studies collectively investigated 793 cases and 1011 controls. Nine studies were conducted on the European continent, while only one study was conducted in Asia (South Korea) [41]. Of the nine studies conducted in Europe, seven were conducted in Turkey [25,26,27,40,41,43,44] and two in Italy [19,35]. All included studies followed a case–control design, except Atik et al. [44] who used a cross-sectional design. Three studies investigated pediatric patients [47], while the remaining studies investigated adult patients (see Table 2).

### 3.3. Homocysteine Meta-Analysis Results

**Figure 2:**
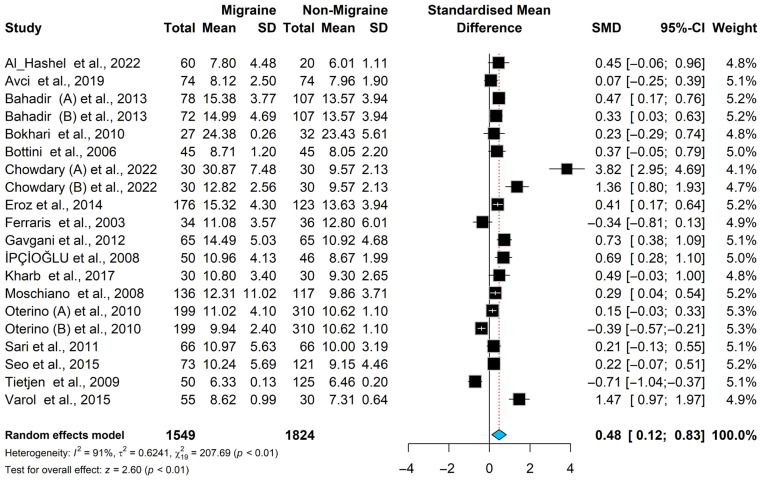
Forest plot for the homocysteine meta-analysis in patients with migraine and controls [17,18,19,29,30,31,32,33,34,35,36,37,38,39,40,41,42].

The pooled standardized mean difference in the levels of homocysteine is significantly higher in the migraine patients compared to the non-migraine controls [SMD = 0.48, 95% CI (0.12; 0.83); *p* < 0.01]; see Figure 2. Higgins’ index (I^2^ = 91.0%) and the Cochrane Q statistic (Cochrane Q = 207.69, *p* < 0.0001) both indicate significant heterogeneity, and the random-effects model was used. To trace possible sources of heterogeneity, we conducted a sensitivity analysis to identify studies with higher heterogeneity that significantly affect the overall estimate. The Baujat plot (Figure S1) and sensitivity analysis did not identify a single study that contributed substantially to the heterogeneity; see Figure S2. Therefore, none of the studies were excluded from the meta-analysis.

#### 3.3.1. Subgroups and Meta-Regression Analysis for Homocysteine

We conducted a subgroup analysis by study continent, dividing studies into two groups: the European countries group and the non-European countries group. The results showed that the European countries group had significantly higher homocysteine levels in cases than in controls [SMD = 0.29; 95% CI (0.04; 0.54); *p* = 0.02; I^2^ = 87.0%]. The non-Europe countries showed the same pattern; however, the *p*-value was borderline [SMD = 0.79; 95% CI (−0.10; 1.68); *p* = 0.08; I^2^ = 94.0%], see Figure S3, Table 3. Another subgroup analysis was conducted, dividing the studies into two groups based on the methods used to assay homocysteine levels: one group used immunoassay methods, and the other used other analytical methods. The results showed that both groups had higher levels of homocysteine in cases of migraine compared to controls; however, the difference was significant in the group using other analytical methods [SMD = 0.28; 95% CI (0.08; 0.49); *p* < 0.01; I^2^ = 84.0%], while it was borderline in the immunoassay group [SMD = 0.68; 95% CI (−0.08; 1.43); *p* = 0.08; I^2^ = 94.0%], see Figure S4, and Table 3. Another subgroup analysis was conducted based on the study design; studies were categorized as case–control or cross-sectional. The findings showed that case–control group had a higher level of Hcy in cases than controls [SMD = 0.26; 95% CI (0.04; 0.48); *p* = 0.02; I^2^ = 87.0%], while it was borderline in the cross-sectional group [SMD = 1.85; 95% CI (−0.10; 3.80); *p* = 0.06; I^2^ = 95.0%], see Table 3 and Figure S5.

In the meta-regression analysis, we investigate numerical covariates such as year of publication, study sample size, and NOS, as well as categorical variables, including geographical continent, and methods of homocysteine assay. The results showed that only the year of publication significantly affects Hcy levels directly [estimation coefficient = 0.087; *p* = 0.017], as shown in Table 4.

#### 3.3.2. Publication Bias for Homocysteine

**Figure 3:**
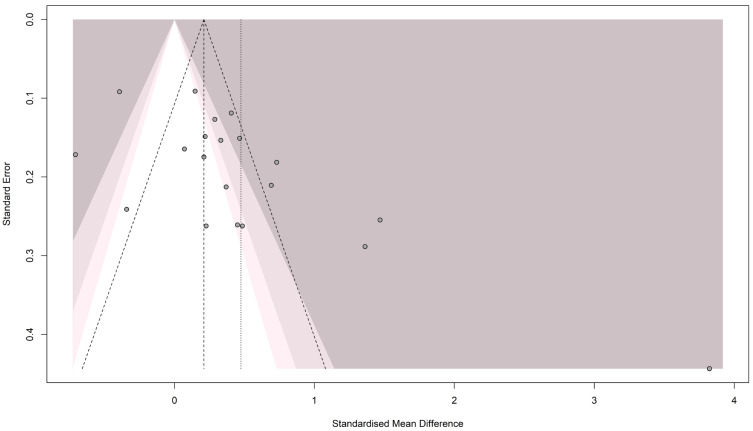
Funnel plot for the Homocysteine meta-analysis in patients with migraine and controls.

**Figure 4:**
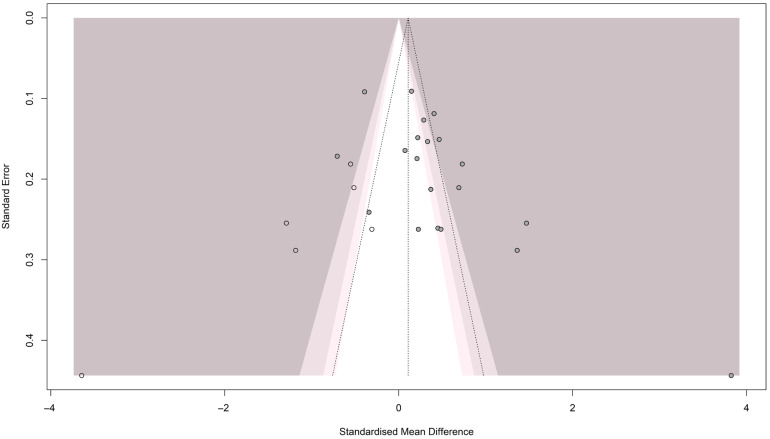
Trim and fill methods for Homocysteine meta-analysis.

The funnel plot showed an asymmetrical pattern across the studies, indicating publication bias (see Figure 3). Egger’s test [t = 3.14; *p* = 0.005] and Begg’s test [z = 2.17; *p* = 0.029] both showed significant results, confirming the presence of publication bias. Accordingly, the trim-and-fill method was applied, adding 6 studies to resolve the funnel plot asymmetry and generate a new pooled estimate [SMD = 0.108; 95% CI (−0.334; 0.550); *p* = 0.632; I^2^ = 93.1%], as shown in Figure 4.

### 3.4. Vitamin B12 Meta-Analysis Results

**Figure 5:**
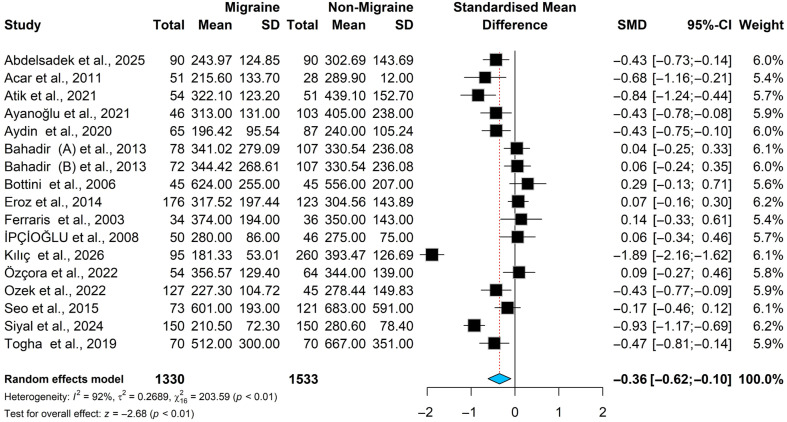
Forest plot for the vitamin B12 meta-analysis in patients with migraine and controls [13,19,22,30,31,32,35,41,43,44,45,46,47,48,49,50].

The pooled standardized mean difference in vitamin B12 levels is significantly lower in migraine patients than in non-migraine controls [SMD = −0.36, 95% CI (−0.62; −0.10); *p* < 0.01] in a random-effects model; see Figure 5. The Higgins’ index (I^2^ = 92.1%) and Cochrane Q (Q = 203.59; *p* < 0.001) both indicate significant heterogeneity, so the random-effects model was used.

To investigate potential sources of heterogeneity, we used a Baujat plot (see Figure S6) to visually assess whether studies with high heterogeneity and greater impact on the effect estimate were included in the meta-analysis. The plot showed that the Kılıç et al. study has the highest heterogeneity and is likely to affect the overall effect estimate. A sensitivity analysis (see Figure S7) was performed, and it shows that deleting Kılıç et al. reduces heterogeneity from 92.3% to 81.0%, while the overall effect estimate does not change significantly [SMD = −0.26, 95% CI [−0.44; −0.07], *p* < 0.010; I^2^ = 81.0%]. However, since the overall heterogeneity remains >50% and is considered significant, we retained the Kılıç et al. study in the meta-analysis.

#### 3.4.1. Subgroup Analysis and Meta-Regression for B12

To conduct the subgroup analysis, we have divided the studies by geographical location into those conducted in Europe and those conducted in the non-European continent. The analysis showed that, the group of Non-Europe continent has a significantly lower levels of B12 compared to controls [SMD = −0.51, 95% CI [−0.83; −0.19], *p* ≤ 0.010; I^2^ = 82.0%], while the Europe continent group showed a borderline significancy SMD = −0.31, 95% CI [−0.64; 0.02], *p* = 0.07; I^2^ = 93.0%]. The heterogeneity level is significant in both groups (see Table 3 and Figure S8). Additionally, subgroup analysis was conducted based on the methods used in the vitamin B12 assay, dividing studies into those using immunoassay techniques and those using other techniques. The results showed that the group used immunoassay techniques has significantly low levels of B12 in cases vs controls [SMD = −0.36, 95% CI (−0.65; −0.08), *p* = 0.01; I^2^ = 83.0%], while no significant difference was observed in other techniques group [SMD = −0.35, 95% CI (−0.76; 0.05), *p* = 0.09; I^2^ = 95.0%], see Table 3 and Figure S9.

Meta-regression analysis was conducted by evaluating numerical covariates, including year of publication, NOS score, and study sample size, in addition to categorical variables such as study continent, study population (adult vs. children), and B12 assay method. The results showed that only the year of publication significantly affects B12 levels inversely [estimate coefficient = −0.77; *p* = 0.046], as shown in Table 4.

#### 3.4.2. Publication Bias Testing for B12

**Figure 6:**
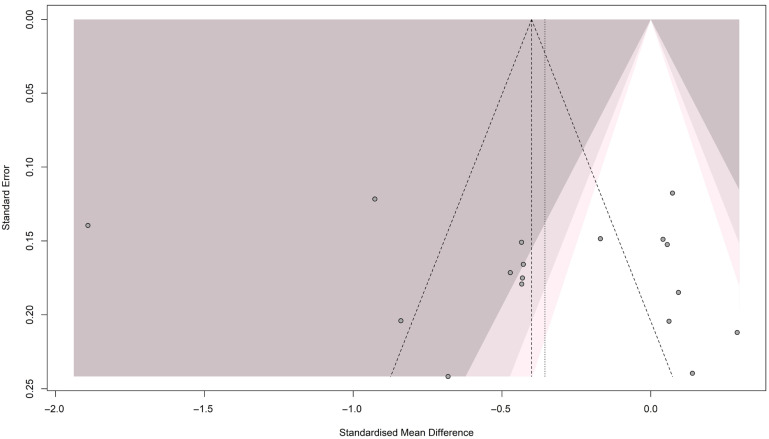
Funnel plot for vitamin B12 meta-analysis in patients with migraine and controls.

To examine for publication bias, we created a funnel plot and looked for visual asymmetry in the distribution of the plotted studies; none was detected (see Figure 6). Further quantitative testing for publication bias was performed using Egger’s test (z = 0.83; *p* = 0.420) and Begg’s test (z = 0.04; *p*-value = 0.967), both of which showed no evidence of publication bias.

### 3.5. Folate Meta-Analysis Results

**Figure 7:**
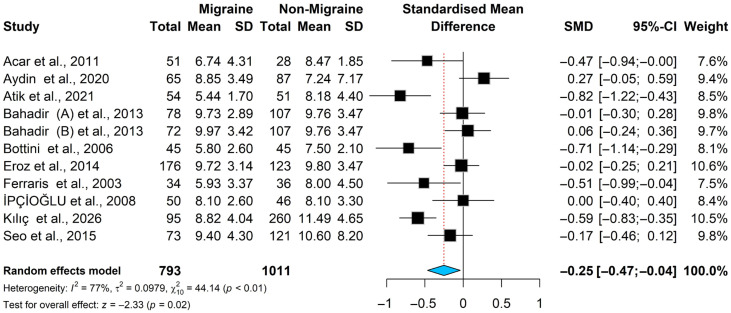
Forest plot for the Folate meta-analysis in patients with migraine and controls [19,30,31,32,35,41,43,44,46,47].

The pooled standardized mean difference in folate levels is significantly lower in migraine patients than in non-migraine controls [SMD = −0.25 [−0.47; −0.04], *p* = 0.02; I^2^ = 77.3%]; see Figure 7. Since heterogeneity is significant in Higgins’ and Cochrane’s Q statistic [Q = 44.14; *p* < 0.0001], we used a random-effects model.

We plot the pooled studies in Baujat plot (see Figure S10), and they showed that Kilic et al., is the only study that has highest heterogeneity and affects the effect size; however, sensitivity analysis revealed that neither deletion of Kilic et al. nor any other study will change the heterogeneity significantly, therefore we proceed with the inclusion of Kilic et al. study, see Figure S11.

#### 3.5.1. Subgroup Analysis and Meta-Regression for Folate

We conducted subgroup analysis by study population, dividing the studies into those investigating pediatric patients and those investigating adult patients. The analysis showed that the adult group had significantly lower levels of folate in cases of migraine compared with controls [SMD = −0.21 [−0.42; −0.00] *p* = 0.05; I^2^ = 64.3%], whereas no significant finding was observed in the pediatrics group [SMD = −0.34 [−0.95; 0.27] *p* = 0.27; I^2^ = 90.0%]; see Table 3 and Figure S12. Another stratification analysis was conducted based on the laboratory methods used in the included studies to estimate folate levels. The studies were categorized into two groups: one used immunoassay methods, and the other used other methods. The analysis showed that both groups reported lower folate levels in cases; however, this difference was not statistically significant (see Table 3 and Figure S13).

Meta-regression analysis revealed that only NOS score [estimation coefficient = −0.918; *p* < 0.001] and those in the European continent group [estimation coefficient = −0.88; *p* = 0.026] were found to inversely affect folate levels, as seen in Table 4.

#### 3.5.2. Publication Bias Testing for Folate

**Figure 8:**
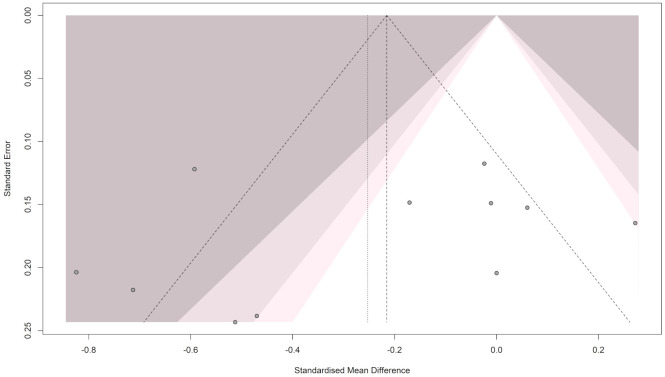
Funnel plot for folate meta-analysis in patients with migraine and controls.

We investigated evidence of publication bias visually and quantitatively. The funnel plot showed no asymmetry among the included studies (see Figure 8). Likewise, both Eager’s test [t = −0.92; *p* = 0.3827] and Begg’s test [z = −0.47, *p*-value = 0.640] were not significant, indicating no publication bias.

### 3.6. Cumulative Meta-Analysis for Homocysteine, B12 and Folate

**Figure 9:**
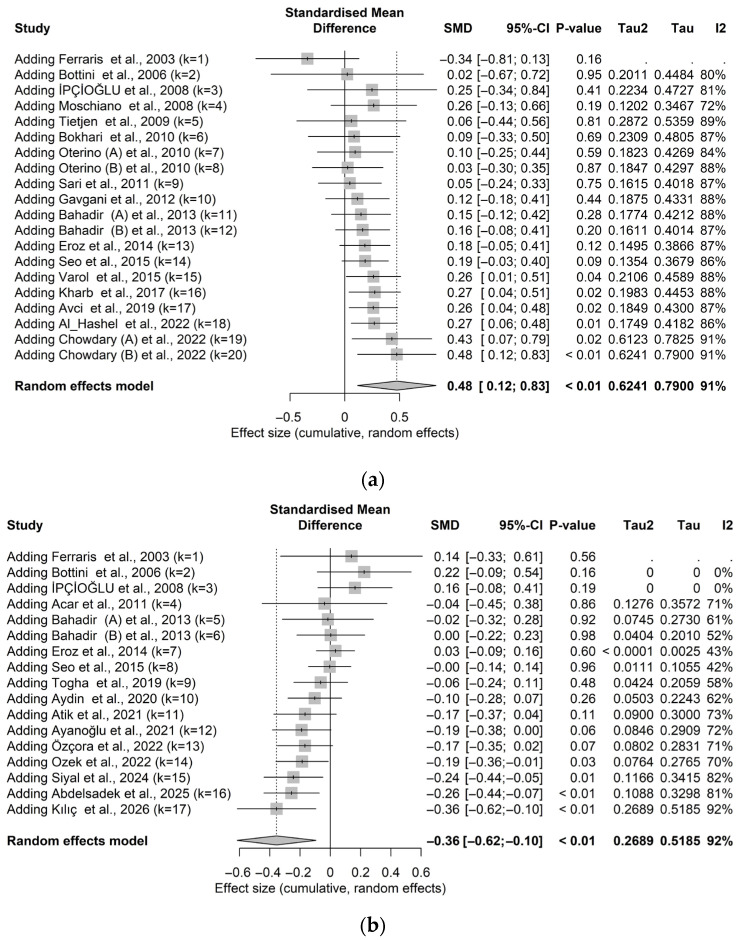

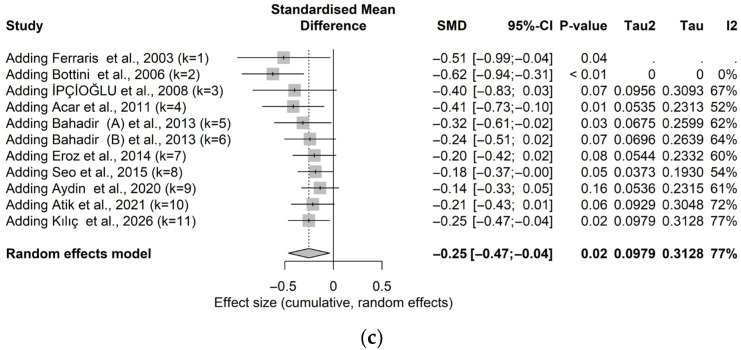
Cumulative meta-analysis for the levels of homocysteine, vitamin B12 and folate in migraine cases and non-migraine controls (**a**) Homocysteine cumulative meta-analysis [17,18,19,29,30,31,32,33,34,35,36,37,38,39,40,41,42]; (**b**) vitamin B12 cumulative meta-analysis [13,19,22,30,31,32,35,41,43,44,45,46,47,48,49,50]; (**c**) folate cumulative meta-analysis [19,30,31,32,35,41,43,44,46,47].

The cumulative meta-analysis of homocysteine demonstrated a progressive accumulation of evidence supporting an increased primary effect in patients with migraine. The final pooled SMD derived from the random-effects model indicated significantly elevated Hcy levels in individuals with migraine. As illustrated in the cumulative forest plot (Figure 9a), the earliest included study reported lower homocysteine levels, contrary to the direction of the overall pooled effect. With the sequential inclusion of subsequent studies, a gradual shift toward higher homocysteine levels in migraine patients was observed, although this trend was initially not statistically significant. Upon incorporation of the 15th study in 2015, the pooled effect size became statistically significant and remained stable through to the most recent study, published in 2022. Heterogeneity remained consistently high throughout the analysis and continued to increase, reaching 91.0% with the inclusion of the latest study.

Similarly, the cumulative meta-analysis of vitamin B12 levels revealed that the initial studies were characterized by wide confidence intervals that crossed the null value, with an overall tendency toward higher vitamin B12 levels in migraine patients (see Figure 9b). This pattern evolved over time, and by the inclusion of the 13th study, the pooled effect estimate approached null. Thereafter, the confidence intervals no longer crossed zero, and the overall effect became consistently statistically significant. The final pooled SMD indicated significantly lower vitamin B12 levels in migraine patients compared with non-migraine controls. Heterogeneity fluctuated across the cumulative analysis, reaching a maximum of 92.0% in the final model.

For folate, the cumulative meta-analysis showed that the sequential addition of studies consistently yielded a pooled SMD, indicating lower levels in migraine patients. The confidence interval of the initial study excluded the null value; however, as additional studies were incorporated, the confidence intervals alternately expanded and narrowed, crossing and excluding the null value intermittently (see Figure 9c). From the 10th study onward, the difference remained consistently significant, with folate levels persistently lower in migraine cases. Heterogeneity was initially absent in the first two studies but subsequently fluctuated across analyses, reaching a peak of 77.0% with the inclusion of the final study.

The significance of this cumulative analysis lies in its demonstration that the observed association is not driven by a single influential study but rather emerges from the progressive accumulation of evidence over time. Despite the persistently high heterogeneity, statistically significant effects were ultimately achieved, underscoring the robustness of the overall association.

### 3.7. GRADE Certainty of Evidence Results

Based on the GRADE certainty assessment, the quality of evidence for the outcome “homocysteine SMD” was rated as low. This rating was primarily driven by substantial heterogeneity among the included studies and evidence of publication bias (see Table 5).

## 4. Discussion

The current meta-analysis aimed to update and synthesize evidence on the association between Hcy, vitamin B12 and folate levels in patients with migraine compared with non-migraine controls. The major finding of this study is that migraine patients exhibit significantly higher serum Hcy levels than controls, irrespective of age group. Also, cases of Hcy-lowering vitamins were significantly lower in migraine cases than in controls. The previous meta-analysis by Limpas and colleagues reported significantly higher Hcy levels and lower folate concentration in migraine patients, but did not observe a significant reduction in vitamin B12 levels [10]. Our meta-analysis incorporated 17 studies, including 1549 cases, compared with the 16 studies totaling 1243 cases in the Limpas et al. study [10]. However, our meta-analysis excluded conference proceedings and gray literature, which were included in the previous meta-analysis. In addition, we performed more in-depth heterogeneity tracing by conducting subgroup analysis based on geographical region, methods used to quantify Hcy, and study designs. Furthermore, meta-regression analysis was conducted to determine whether quantitative covariates, such as sample size, year of publication, and Newcastle–Ottawa Scale (NOS) score, in addition to other categorical variables, affected the estimated effect size. Furthermore, a cumulative meta-analysis was also conducted to chronologically trace the evolution of the generated evidence. Finally, the overall evidence certainty for homocysteine levels was graded by GRADEpro tools. Collectively, these methodological refinements provide an updated assessment of the association between Hcy, vitamin B12, folate and migraine.

In this study, Hcy levels were significantly higher in migraine cases than in non-migraine controls. This trend persisted in subgroup analyses by study continent, particularly among studies conducted in European countries. This observation may be partially explained by genetic susceptibility, as migraine is known to have a strong genetic component and familial pattern [51]. Among these genetic factors are variants in the MTHFR, which are documented to impact Hcy levels [52]. Interestingly, the MTHFR variant C677T (rs1801133) is most prevalent among individuals of European ancestry, followed by East Asian populations [53]. In addition to genetic factors, dietary intake is considered a major determinant of circulating Hcy, vitamin B12 and folate [54]. It has been reported that 59% of adolescents and 58% of adults had very low folate intake in a European country [55]. Furthermore, the multi-center HELENA study, conducted across ten European countries, showed that 50% of the adolescents had insufficient folate levels [56]. In non-European countries, the Hcy levels were also higher in migraine cases compared with non-migraine controls but did not reach statistical significance. Most of the countries in this group were South Asian countries, including Pakistan and India. In Pakistan, vitamin B12 deficiency is reported in 52.4% of the general population [57], while in India it is 48.3% among women of reproductive age [58]. This high prevalence of vitamin B12 deficiency is owing to the predominant vegetarian dietary pattern in these regions [59]. Vegetarianism has been associated with a 4.4 times increased risk of vitamin B12 deficiency compared with non-vegetarian diets [60].

Additionally, subgroup analysis stratified by Hcy quantification method revealed that studies using methods other than immunoassays had significantly higher Hcy levels. From an analytic perspective, HPLC has been shown to detect lower Hcy concentrations with greater sensitivity than immunoassay-based techniques, including the fluorescence polarization immunoassay and chemiluminescence immunoassay [61]. This methodological variability explained some of the inter-study heterogeneity. Likewise, studies using a case–control design demonstrated elevated Hcy levels in migraine patients; however, we believe that this may be due to the number of studies included in the case–control group rather than a true study design effect. Most studies were case–control studies, whereas only two studies used a cross-sectional design. In the meta-regression analysis, only the year of publication positively affected the effect size, among the other investigated covariates. Perhaps this is due to advances in practical methods and clinical practices in recent years.

Pathophysiologically, the association between Hcy and migraine can be attributed to homocysteine’s signaling activity. It has been reported that exposure to high levels of Hcy acts as a potent agonist of N-methyl-D-aspartate (NMDA) receptors, leading to sustained calcium influx into neuronal mitochondria and initiating a cascade of reactions associated with oxidative stress that promote neuronal damage. Such damage lowers the threshold for neuronal depolarization and facilitates cortical spreading depolarization, which is thought to be a main driving mechanism in inducing migraine headaches [62,63,64]. Additionally, the effect of HHcy-induced oxidative stress is not limited to neuronal cells; it extends to the neurovascular system by interfering with NO synthase enzyme activity, thereby reducing NO bioavailability, decreasing neurovascular reactivity, and ultimately leading to neurovascular dysfunction [65,66]. From another perspective, quinolinic acid (QUIN), a metabolite of the Kynurenine pathway, can induce the NO synthase enzyme, generating more NO and promoting nitrosative stress, which, in turn, causes neuronal damage and initiates excitatory signals that precipitate a migraine attack [67]. Both QUIN and Hcy act as NMDA receptor agonists, which may induce excitatory signals and cortical spreading associated with migraine attacks [68].

Vitamin B12 and folate are both needed as co-enzymes for the conversion of homocysteine to methionine, a reaction catalyzed by the methionine synthase enzyme. Deficiency of either vitamin disrupts the entire reaction, leading to HHcy [69]. Apart from HHcy, deficiencies in vitamin B12 or folate also affect the availability of S-adenosyl methionine, a universal methyl group donor whose reactions include DNA methylation, epigenetic regulation, and neurotransmitter metabolism [70]. Impairment of S-adenosyl methionine may disrupt all these vital reactions, which have been implicated in susceptibility to migraine [71]. From another perspective, isolated vitamin B12 deficiency can contribute to migraine pathophysiology. Vitamin B12 is a coenzyme for the mitochondrial enzyme methyl-malonyl CoA mutase reaction, which is needed in the conversion of methyl-malonyl Co-A to succinyl-CoA, an intermediate in the energy production pathway and the citric acid cycle [69]. In the case of B12 deficiency, accumulation of methylmalonyl-CoA, in addition to a reduction in energy production, will cause mitochondrial dysfunction and axonal injury [72]. Mitochondrial dysfunction is reported to contribute to migraine pathogenicity [73].

In the literature, there are many sources of B12 deficiency and HHcy, among them is diabetes mellitus. It has been reported that diabetic renal complications may interfere with the clearance of Hcy and precipitate HHcy [74,75]. Additionally, chronic usage of metformin in type 2 diabetes mellitus interferes with the absorption of B12 from the intestine and precipitates HHcy [76,77]. In the current meta-analysis, diabetes mellitus is excluded in most of the included studies [29,30,32,33,39,42,44]; some studies did not state such exclusions. In addition to metformin, other drugs such as proton pump inhibitors [78] and contraceptive pills [79] may reduce B12 and folate levels, respectively. Few studies in the current meta-analysis explicitly stated the exclusion of these drugs [36].

Experimental clinical trials demonstrated that supplementation with B12 and folate for 4 months resulted in a significant reduction in migraine attack frequency, improved disability scores, and reduced use of abortive drugs [80]. Another trial with folate and B6 showed a similar beneficial outcome [21]. A further trial demonstrated that supplementation with B12, folate, and B6 in patients with a genetic risk of hyperhomocysteinaemia not only improved migraine symptoms but also decreased Hcy levels by 39.0%, with a reduction of nearly 4 µmol/L [15]. All these premises support the beneficial roles of vitamin B12 and folate as well as the association of HHcy with migraine.

To place these findings within a clinical context, we back-transformed our SMD = 0.48, 95% CI = (0.12, 0.83) to an absolute mean difference (MD) [this is done by using the SD of 4 μmol/L [81] as a reference range for adults subjects, who are the majority in the current study], which yielded an MD of 1.92 μmol/L, 95% CI = (0.48, 3.32). This estimate is almost the same as 1.64 μmol/L (95% CI = 0.80, 2.44), which was reported by Limpas et al.’s study [10]. In clinical terms, this reduction sounds marginal; however, in another neurovascular disease, such as cases of cerebral small vessel disease, the SMD of Hcy was 0.50, 95% CI = (0.36, 0.64) [82], and after transformation it corresponds to 2.0 μmol/L 95% CI = (1.44, 2.56), which is exactly our absolute mean difference. Similarly, in cases of recurrent cerebral stroke, the mean difference in Hcy was 1.2 μmol/L (95% CI = (1.05, 2.3)) [83], which is within our range. Furthermore, lowering Hcy by 3 μmol/L has been shown to be cardioprotective [84]. Yet other trials showed that a small reduction in Hcy levels is associated with a minor clinical effect, especially for cardiovascular events [85]. Taken together, the observed SMD in the current study represents a moderate estimated difference and is graded as of low certainty, yet previous studies showed a similar difference range with acceptable clinical outcomes.

The present study had some limitations to be considered. First, although we conducted subgroup and meta-regression analyses, the heterogeneity remained substantial. Second, dietary intake, an important determinant of Hcy levels and vitamin status, is largely under-reported in the included studies. Third, personal factors such as smoking and alcohol consumption, in addition to medication usage such as metformin, proton pump inhibitors and others that are known to interfere with B12 or folate bioavailability, were not excluded in the majority of the studies. Fourth, the methods used to measure homocysteine, vitamin B12, and folate varied across studies, including differences in analytical procedures, cutoff values, and inter-laboratory standardization. As these methodological details were not consistently reported, they remain a significant potential source of heterogeneity. Finally, publication bias was detected in homocysteine analysis, yet was adjusted for using trim-and-fill methods, and the previous meta-analysis reported the same [10]. Nonetheless, the present study has several strengths. We searched multiple databases and retrieved more original articles than in the previous meta-analysis, allowing a comprehensive synthesis of the available literature. Moreover, a cumulative meta-analysis was conducted for the first time on this topic, which traces back the generation of the present evidence chronologically.

## 5. Conclusions

In conclusion, the current updated meta-analysis demonstrates an association between elevated Hcy levels, and decreased levels of vitamin B12 and folate in adult and pediatric patients with migraine. These findings suggest that neurologists and pediatricians may evaluate and adjust Hcy, vitamin B12, and folate levels in migraine patients. Although a biochemical relationship among these biomarkers is established, this meta-analysis does not establish whether decreased B12 and folate levels directly contribute to increased homocysteine in individuals with migraine. Further longitudinal studies that account for dietary intake, personal habits, and drug history are needed to establish a causal relationship.

## Figures and Tables

**Table 1:** The PICOS criteria used in the current systematic review and meta-analysis.

Element	Description
P (Population)	Adults, children
I (Intervention)	Homocysteine OR Hcy
	B12 OR vitamin B12 OR cyanocobalamin OR cobalamin OR methylcobalamin OR mecobalamin
	Folic acid OR folate OR vitamin B9
C (Comparison)	Healthy control OR non-migraine OR non-headaches OR normal control
O (Outcome)	Migraine patients OR migraine with aura OR migraine without aura
S (Study design)	Case–control OR cross-sectional OR cohort

**Table 3:** Subgroup analysis of the homocysteine, vitamin B12 and folate levels in association with migraine.

Subgroup	Number of Studies	Number of Migraine	Number of Non-Migraine	SMD (95% CI)	*I*^2^ Index
Homocysteine subgroups					
**Continents**					
Europe	10	1184	1371	0.29 (0.04; 0.54) *	87.0%
Non-Europe	7	365	453	0.79 (−0.10; 1.68)	94.0%
**Hcy assay**					
Immunoassay	9	574	660	0.68 (−0.08; 1.43)	94.0%
Others	8	975	1164	0.28 (0.08; 0.49) *	84.0%
**Study Design**					
Case–control	15	1429	1744	0.26 (0.04; 0.48) *	87.0%
Cross-sectional	2	120	80	1.85 (−0.10; 3.80)	95.0%
**Vitamin B12 subgroups**					
**Continents**					
Europe	12	947	1102	−0.31 (−0.64; 0.02)	93.0%
Non-Europe	4	383	431	−0.51 (−0.83; −0.19) *	82.0%
**B12 assay**					
Immunoassay	7	551	557	−0.36 (−0.65; −0.08) *	83.0%
Others	9	779	976	−0.35 (−0.76; 0.05)	95.0%
**Folate subgroups**					
**Study group**					
Adult	7	588	619	−0.21 (−0.42; −0.00) *	64.3%
Children	3	205	392	−0.34 (−0.95; 0.27)	90.0%
**Folate assay**					
Immunoassay	3	174	195	−0.18 (−0.39; 0.03)	12.0%
Others	7	619	816	−0.28 (−0.56; 0.01)	83.0%

* Statistical significance; Hcy: homocysteine; SMD: standardized mean difference; 95% CI: 95% confidence interval; Variables used for subgroup analysis are shown in bold.

**Table 4:** Meta-regression analysis of the homocysteine, vitamin B12 and folate covariates.

	Estimation Coefficient	Standard Error	*p*-Value	95% CI
**Co-variates For Hcy**				
**NOS**	0.0688	0.2852	0.8093	(0.2852; 0.8093)
**Year of publication**	**0.0874**	**0.0368**	0.0175 *	(0.0368; 0.0175)
**Sample_size**	−0.0013	0.0014	0.353	(0.0014; 0.353)
**Hcy_assay**				
Immunoassay	Reference	Reference		
Others	−0.1417	0.3709	0.7024	(0.3709; 0.7024)
**Continent**				
Europe	Reference	Reference		
Non-Europe	−0.1962	0.4385	0.6545	(0.4385; 0.6545)
**Co-variates For vitamin B12**				
**NOS score**	0.10	0.253	0.671	(0.253; 0.671)
**Year of publication**	**−0.77**	**0.389**	**0.046**	**(0.389; 0.046)**
**Hcy assay method** Immunoassay Others	Reference 0.07	Reference 0.253	0.757	(0.253; 0.757)
**Sample_size**	−0.002	0.002	0.274	(0.002; 0.274)
**Continent** Europe Non_Europe	Reference −0.47	Reference 0.436	0.278	(0.436; 0.278)
**Co-variates For Folate**				
**NOS**	**−0.9186**	**0.2309**	**0 <** 0.0001 *	**(0.2309; 0.0001)**
**Year of Publication**	−0.0019	0.0195	0.9225	(0.0195; 0.9225)
**Study Group** Adult Children	Reference −0.0209	Reference 0.2036	0.9182	(0.2036; 0.9182)
**Sample sizes**	−0.0026	0.0015	0.0823	(0.0015; 0.0823)
**Hcy_assay** Immunoassay Others	Reference 0.095	Reference 0.2516	0.7058	(0.2516; 0.7058)
**Continent** Non_Europe Europe	Reference **−0.8807**	Reference **0.3955**	0.026 *	**(0.3955; 0.026)**

95% CI: 95% confidence interval; NOS: Newcastle–Ottawa Scale. Bold font indicates statistically significant finding. * *p* < 0.05.

**Table 5:** GRADE table association of homocysteine levels in migraine patients compared with non-migraine controls.

Quality Assessment							Summary of Findings	
No. of Patients with Migraine/Non-Migraine (No. of Studies)	Study Design	Risk of Bias	Inconsistency	Indirectness	Imprecision	Publication Bias	Overall Quality of Evidence	Comment
1549/1824 (17 studies)	Observational studies	Not serious	Not serious	Not serious	Not serious	Publication bias strongly suspected ^a,b^	⨁⨁◯◯ Low	SMD: −0.48; (95% CI = 0.12, 0.83)

GRADE working group grades of evidence. Moderate quality: further research is likely to have an important impact on our confidence in the estimate of effect and may change the estimate. SMD: standardized mean difference. ^a^ There is evidence of publication bias after applying Egger’s test. ^b^ Trim and fill method was used, and 6 more studies were added to correct funnel plot asymmetry.

## Data Availability

The original contributions presented in this study are included in the article/Supplementary Material. Further inquiries can be directed to the corresponding author.

## Supplementary Materials

The following supporting information can be downloaded at: https://www.mdpi.com/article/10.3390/brainsci16020218/s1. Table S1. Prisma Checklist. Table S2. Searching strategies for PubMed, Google Scholar, ScienceDirect, and the Cochrane Library. Figure S1. Baujat plot for studies pooled in Homocysteine meta-analysis in patients with migraine and controls [17,18,19,29,30,31,32,33,34,35,36,37,38,39,40,41,42]. Figure S2. Sensitivity analysis for studies pooled in Homocysteine meta-analysis in patients with migraine and controls [17,18,19,29,30,31,32,33,34,35,36,37,38,39,40,41,42]. Figure S3. Subgroup analysis based on geographical continent for studies pooled in Homocysteine meta-analysis in patients with migraine and controls [17,18,19,29,30,31,32,33,34,35,36,37,38,39,40,41,42]. Figure S4. Subgroup analysis based on Homocysteine quantification methods for studies pooled in Homocysteine meta-analysis in patients with migraine and controls [17,18,19,29,30,31,32,33,34,35,36,37,38,39,40,41,42]. Figure S5. Subgroup analysis based on study designs for studies pooled in Homocysteine meta-analysis in patients with migraine and controls [17,18,19,29,30,31,32,33,34,35,36,37,38,39,40,41,42]. Figure S6. Baujat plot for studies pooled in vitamin B12 meta-analysis in patients with migraine and controls [13,19,22,30,31,32,35,41,43,44,45,46,47,48,49,50]. Figure S7. Sensitivity analysis for studies pooled in vitamin B12 meta-analysis in patients with migraine and controls [13,19,22,30,31,32,35,41,43,44,45,46,47,48,49,50]. Figure S8. Subgroup analysis based on geographical location for studies pooled in vitamin B12 meta-analysis in patients with migraine and controls [13,19,22,30,31,32,35,41,43,44,45,46,47,48,49,50]. Figure S9. Subgroup analysis based on methods used to quantify vitamin B12 for studies pooled in vitamin B12 meta-analysis in patients with migraine and controls [13,19,22,30,31,32,35,41,43,44,45,46,47,48,49,50]. Figure S10. Baujat plot for studies pooled in Folate meta-analysis in patients with migraine and controls [19,30,31,32,35,41,43,44,46,47]. Figure S11. Sensitivity analysis for studies pooled in Folate meta-analysis in patients with migraine and controls [19,30,31,32,35,41,43,44,46,47]. Figure S12. Subgroup analysis based on study group for studies pooled in Folate meta-analysis in patients with migraine and controls [19,30,31,32,35,41,43,44,46,47]. Figure S13. Subgroup analysis based on methods used to quantify folate for studies pooled in folate meta-analysis in patients with migraine and controls [19,30,31,32,35,41,43,44,46,47].

## Abbreviations

DALYs	Disability-adjusted life years
EIA	Enzyme immunoassay
ELISA	Enzyme-linked immunoassay
Hcy	Homocysteine
HELENA	Nutrition and lifestyle in European adolescents
HHcy	Hyperhomocysteinemia
ICHD	International classification of headache disorders
IHS	International Headache Society
MA	Migraine with aura
HPLC	High-performance liquid chromatography
MeSH	Medical subject heading
MTHFR	Methylenetetrahydrofolate reductase
NMDA	N-methyl-D-aspartate
NOS	Newcastle–Ottawa scale
PICOS	Population Intervention Comparison Outcome and Study design
PRISMA	Preferred Reporting Items for Systematic Reviews and Meta-Analyses
QUIN	Quinolinic acid
SD	Standard deviation
SMD	Standardized mean difference
YLDs	Years lived with disability
YLLs	Years of life lost
